# Age‐Specific Clinical Biomarker Ranges in Acute Head Injury, Non‐TBI Trauma, and Healthy Control Subjects in the Emergency Department

**DOI:** 10.1111/acem.70298

**Published:** 2026-04-28

**Authors:** Katherine D. Mayes, Timothy E. Van Meter, Nazanin Mirshahi, Sally Boyd, Danielle Sandsmark, Katya Rascovsky, Ramon Diaz‐Arrastia, Justin Weppner, W. Frank Peacock, Damon R. Kuehl, the HeadSMART II Investigators

**Affiliations:** ^1^ Department of Emergency Medicine Virginia Tech Carilion School of Medicine Roanoke Virginia USA; ^2^ BRAINBox Solutions, Inc Richmond Virginia USA; ^3^ Department of Neurology University of Pennsylvania Perelman School of Medicine Philadelphia Pennsylvania USA; ^4^ Department of Internal Medicine Virginia Tech Carilion School of Medicine Roanoke Virginia USA; ^5^ Department of Emergency Medicine Baylor College of Medicine Houston Texas USA

## Abstract

**Objectives:**

Blood‐based biomarkers for traumatic brain injury (TBI) are increasingly integrated into diagnostic algorithms, but their interpretation may be confounded by age‐related neurological changes. This study quantified the relative effects of age and TBI on biomarker concentrations to determine whether age‐related variation approaches or exceeds that associated with injury.

**Methods:**

Serum biomarkers were analyzed from 762 adults enrolled in the HeadSMART II and HeadSMART Geriatric studies, including healthy controls (*n* = 88), non‐head trauma controls (*n* = 99), and mild TBI patients (GCS 13–15, *n* = 575). Participants were categorized by age (18–40, 41–64, 65–74, ≥ 75 years). Six TBI‐relevant biomarkers (glial fibrillary acidic protein [GFAP], brain‐derived neurotrophic factor [BDNF], neurogranin [NRGN], α‐synuclein [SNCA], suppression of tumorigenicity 2 [ST2], and von Willebrand factor [vWF]) were quantified using validated immunoassays (BRAINBox Solutions). Biomarker levels were compared using two‐way ANOVA, and the relative effects of age and injury were estimated using Cohen's *f*.

**Results:**

Age significantly influenced several biomarkers. GFAP showed strong age‐related increases, with significant elevations across age strata (*p* < 0.001), exceeding the effect of head injury alone. vWF also increased significantly with age (*p* < 0.001), while ST2 did not show a main effect of age (*p* = 0.404), although age interacted with group (*p* < 0.001). SNCA demonstrated modest age effects (*p* = 0.001), particularly in older trauma and TBI participants. NRGN showed no significant age‐related changes (*p* = 0.454), and BDNF exhibited age effects within interaction terms (*p* < 0.001). Overall, age‐associated effect sizes for GFAP and vWF were comparable to, or greater than, those of head injury.

**Conclusions:**

Age exerts substantial influence on circulating biomarker concentrations, particularly GFAP and vWF, often rivaling or exceeding TBI‐related changes. Diagnostic algorithms that fail to adjust for age may risk misclassification, especially among older adults, underscoring the need for age‐normalized biomarker interpretation.

## 1 Background

Traumatic brain injury (TBI) remains a leading cause of death and disability worldwide, with clinical presentations ranging from mild transient symptoms to life‐threatening neurological compromise [1]. Outcomes are highly variable, and some patients with seemingly mild injuries experience lifelong impairment, while others recover fully from apparent severe trauma. Despite its prevalence, TBI continues to pose significant diagnostic challenges [2]. These challenges stem from heterogeneity in injury mechanisms, overlapping symptom profiles, and the limitations of traditional diagnostic tools, which often fail to detect subtle or evolving injury, especially in cases of mild or repetitive trauma [3]. One study found that the ability of Emergency Department (ED) physicians to prospectively identify patients who would have mTBI symptoms at 90‐days is very poor (sensitivity 8.1%, specificity 54.5%) [4]. Standard imaging techniques such as CT scans lack sensitivity for microscopic or diffuse injury, and clinical assessments can be influenced by confounding factors such as intoxication, baseline cognition, and concurrent injuries [5].

To address these limitations, recent efforts have turned toward multidimensional diagnostic frameworks. The Clinical, Biomarker, Imaging–Modifiers (CBI‐M) model integrates clinical evaluation, blood‐based biomarkers, and neuroimaging to improve diagnostic precision and individualized risk stratification [6]. Among these advances, the recent FDA clearance of glial fibrillary acidic protein (GFAP) and ubiquitin C‐terminal hydrolase‐L1 (UCH‐L1) assays for TBI triage has underscored the clinical potential of biomarkers to complement imaging [7].

Circulating biomarker levels are influenced not only by injury but also by age‐related changes and comorbid conditions affecting neurological, vascular, hepatic, and renal function [8, 9, 10]. Without accounting for these influences, biomarker interpretation can be misleading, as both aging and chronic disease can alter baseline concentrations independent of acute trauma [11, 12].

Several biomarkers of neurologic and vascular health, including brain‐derived neurotrophic factor (BDNF), GFAP, neurogranin (NRGN), α‐synuclein (SNCA), suppression of tumorigenicity 2 (ST2), and von Willebrand factor (vWF), demonstrate measurable variation across the lifespan [9, 12]. For example, BDNF typically declines with age and neurodegenerative disease, while GFAP and vWF increase with gliosis and vascular remodeling [13]. SNCA fluctuates with both aging and Lewy body pathology, and ST2 reflects systemic inflammation common in older adults [14, 15]. NRGN, a marker of synaptic integrity, can decrease with age‐related synaptic loss independent of injury [16].

These patterns raise a critical question: are the effects of TBI on these biomarkers large compared to the effects of normal aging, or are they of similar magnitude? If age exerts an equal or greater influence than trauma, biomarker thresholds derived from younger, healthier populations may not generalize to older adults, risking diagnostic misclassification. Elevated baseline levels in older individuals could mimic TBI, while high pre‐injury variability could obscure true injury‐related increases.

To move toward precision diagnostics, it is essential to quantify the relative magnitude of biomarker changes due to age versus TBI. Our purpose was to evaluate the potential TBI biomarkers of BDNF, GFAP, NRGN, SNCA, ST2, and vWF in cohorts of healthy controls, non‐TBI trauma controls, and head‐injured patients to determine whether the effects of TBI are distinct from or small relative to age‐related variation. By establishing how strongly age influences each biomarker, we aim to clarify which markers require age‐adjustment within diagnostic algorithms to improve clinical accuracy across the adult lifespan.

## 2 Methods

### 2.1 Study Design and Participants

This analysis included participants from two related prospective observational studies, HeadSMART II and HeadSMART Geriatrics, both conducted at Level I trauma centers using identical recruitment procedures and standardized blood collection protocols [17]. Adults aged 18 years and older were eligible if they or a legally authorized representative could provide informed consent and a blood sample within 96 h of injury. Participants were classified into three groups: (1) the *traumatic brain injury (TBI)* cohort, consisting of individuals who sustained blunt head trauma with either clinical signs and symptoms consistent with TBI or CT‐ or MRI‐confirmed intracranial abnormalities, (2) *non‐TBI trauma controls (TC)*, who sustained blunt trauma to body regions other than the head (e.g., orthopedic or soft‐tissue injuries) without clinical suspicion of brain injury, serving as a comparison group for systemic trauma effects; and (3) *healthy controls (HC)*, an ED‐based sample of friends, siblings, and family members of ED patients, with no history of TBI within the preceding 6 months and no active cancer treatment, no recent blood transfusion, and no known neurologic disease.

### 2.2 Inclusion and Exclusion Criteria

Inclusion and exclusion criteria followed those outlined in HeadSMART II [17]. Participants were excluded if they presented with a Glasgow Coma Scale < 13, required general anesthesia at presentation, or were unable to complete study assessments due to cognitive or physical limitations. Additional exclusions included prior head trauma requiring medical attention within the past 6 months; history of disabling stroke, intracranial surgery, brain tumor, seizure disorder, or intracerebral hemorrhage; receipt of chemotherapy or radiation therapy within the past year; dementia requiring assistance with activities of daily living; or psychiatric hospitalization within 90 days. Healthy controls were further excluded for any recent head trauma, internal injuries requiring hospitalization, or other medical conditions precluding safe blood draw or participation. Blood samples for TBI and trauma control participants were collected at the time of ED presentation and within 96 h of injury; healthy control samples were collected at enrollment.

### 2.3 Age Stratification

To evaluate age‐related biomarker variation, participants were stratified into four predefined age groups: 18–40 (younger), 41–64 (middle‐aged), 65–74 (early elderly), and 75–100 years (late elderly). This structure permitted analysis of biomarker trajectories across the adult lifespan and quantification of age‐related effect sizes relative to those of TBI.

### 2.4 Biomarker Quantification

Serum biomarkers were selected for their biologic relevance to neuronal, astrocytic, synaptic, vascular, and inflammatory processes. GFAP, NRGN, BDNF, vWF, and SNCA were quantified using custom‐validated immunoassays developed by BRAINBox Solutions Inc. (Richmond, VA) and run on the Meso Scale Discovery (MSD) QuickPlex 120 electrochemiluminescence immunoassay platform. ST2 was measured using an FDA‐cleared colorimetric ELISA on a SpectraMax M4 multifunction plate reader (Molecular Devices). All samples were run in duplicate, with 80%–120% recovery. Mean coefficients of variation were < 10% for analyte concentrations falling within the quantitative range of the assays. Detailed assay parameters are summarized in Table 1.

**TABLE 1:** Immunoassay performance parameters.

Assay	LLOD^a^	LLOQ^a^	ULOQ^a^
BDNF	0.017	0.330	240
GFAP	0.002	0.008	12
NRGN	0.007	0.013	120
SNCA	0.083	0.823	600
ST2	1.31	2.35	200
vWF	0.454	54.9	40,000

Abbreviations: LLOD, lower limit of detection, calculated as the mean signal × 2.5 times the standard deviation of the 0 standard mean signal; LLOQ, lower limit of quantitation, calculated as the mean signal × 10 times the standard deviation of the 0 standard; ULOQ, upper limit of quantitation, concentration of the top calibrator.

^a^ All values are given in ng/mL.

### 2.5 Statistical Analysis

Statistical analyses were performed using R (version 4.4.2). Biomarker levels were analyzed to assess the effects of study group and age using a factorial design. Biomarker concentrations were log‐transformed (Base 2) to improve normality and stabilize variance. A two‐way analysis of variance (ANOVA) was performed using a linear model that included the main effects of group and age, as well as their interaction, to evaluate whether the relationship between age and the biomarker differed across groups. Model assumptions were thoroughly assessed: homoscedasticity was evaluated by plotting residuals against fitted values, and normality of residuals was examined using Q–Q plots and histograms. Interaction effects were further explored through graphical visualization to illustrate potential age‐by‐group differences in the biomarker levels. Post hoc pairwise comparisons were conducted using estimated marginal means for both group and age, with *p*‐values adjusted for multiple comparisons using the Benjamini‐Hochberg false discovery rate procedure. Finally, effect sizes were calculated using Cohen's *f* to provide a measure of the magnitude of the observed effects.

## 3 Results

A total of 762 participants met all inclusion and none of the exclusion criteria, comprising 88 healthy controls (HC), 99 non‐TBI trauma controls (TC), and 575 TBI group participants. Demographics are summarized in Table 2.

**TABLE 2:** Demographic characteristics.

Variable	Healthy (*n* = 88)	Trauma control (*n* = 99)	Blunt head trauma (*n* = 575)	Total (*n* = 762)	*p*
Age (median, IQR)	45.0 (26.8–63.0)	58.0 (40.5–70.0)	45.0 (28.0–62.0)	48.0 (29.0–64.0)	**< 0.001**
Sex					0.77
Female	46 (52.3%)	48 (48.5%)	277 (48.2%)	371 (48.7%)	
Male	42 (47.7%)	51 (51.5%)	298 (51.8%)	391 (51.3%)	
Race					**< 0.001**
White	63 (71.6%)	65 (65.7%)	325 (56.5%)	453 (59.4%)	
African American/Black	3 (3.4%)	28 (28.3%)	136 (23.7%)	167 (21.9%)	
Asian	9 (10.2%)	3 (3.0%)	10 (1.7%)	22 (2.9%)	
American Indian/Alaskan	0 (0.0%)	0 (0.0%)	4 (0.7%)	4 (0.5%)	
Multiracial	2 (2.3%)	2 (2.0%)	10 (1.7%)	14 (1.8%)	
Not Reported	9 (10.2%)	1 (1.0%)	73 (12.7%)	83 (10.9%)	
Unknown	2 (2.3%)	0 (0.0%)	16 (2.8%)	18 (2.4%)	
Missing	0 (0.0%)	0 (0.0%)	1 (0.2%)	1 (0.1%)	
Ethnicity					**0.007**
Hispanic or Latino	26 (29.5%)	11 (11.1%)	116 (20.2%)	153 (20.1%)	
Not Hispanic or Latino	62 (70.5%)	88 (88.9%)	459 (79.8%)	609 (79.9%)	
Education					**< 0.001**
High school or less	6 (6.8%)	29 (29.3%)	237 (41.3%)	272 (35.7%)	
Some college	58 (65.9%)	58 (58.6%)	290 (50.4%)	406 (53.3%)	
Graduate or professional degree	24 (27.3%)	11 (11.1%)	46 (7.9%)	81 (10.6%)	
Missing	0 (0.0%)	1 (1.0%)	2 (0.3%)	3 (0.4%)	
Participate in organized sports					0.19
No	77 (87.5%)	94 (94.9%)	526 (91.5%)	697 (91.5%)	
Yes	11 (12.5%)	5 (5.1%)	49 (8.5%)	65 (8.5%)	

*Note:* Significance was set at *p* < 0.05.

### 3.1 Sex

Sex distribution was balanced across groups, with no statistically significant differences (*p* = 0.77). Females comprised 52.3% of the healthy controls, 48.5% of the trauma controls, and 48.2% of the TBI group, resulting in an overall sample that was 48.7% female and 51.3% male.

### 3.2 Racial Composition

All cohorts were predominantly White, ranging from 71.6% in the HC group to 56.5% in the TBI group. The proportion of African American/Black participants differed significantly across groups (*p* < 0.001), representing 3.4% of HC, 28.3% of TC, and 23.7% of TBI participants. Asian representation was low across all cohorts (< 3%) and not statistically meaningful. These differences likely reflect both recruitment sources and known disparities in injury epidemiology and healthcare access.

### 3.3 Educational Status

Educational attainment also varied significantly (*p* < 0.001). HCs had the highest education levels, with 27.3% holding graduate or professional degrees, compared with 11.1% of TC and 7.9% of TBI participants. Conversely, 41.3% of TBI patients had a high school education or less, compared with 29.3% of TC and only 6.8% of HC. The overrepresentation of highly educated individuals in the HC group likely reflects the convenience sampling strategy used for community recruitment.

### 3.4 Employment Status

Employment status differed modestly across groups (*p* = 0.042), largely driven by higher rates of retirement among trauma controls and TBI participants, consistent with their older median ages. There were no significant differences in sex distribution, marital status, or participation in organized sports across cohorts.

### 3.5 Injury Mechanism

Injury characteristics for TC and TBI participants are shown in Table 3. Among TBI cases, the most frequent mechanism was falls (40.9%), followed by motor vehicle collisions (26.6%) and assaults (7.5%). Mechanisms differed significantly by age (*p* < 0.001): falls predominated in older adults (56.7% in early elderly vs. 76.2% in late elderly), whereas motor vehicle collisions were most common in younger and middle‐aged adults (47.1% in middle‐aged vs. < 10% in elderly). Assault‐related injuries were concentrated in young adults (12.5%).

**TABLE 3:** Age‐related differences in injury features.

	Trauma control (*n* = 99)	TBI‐young adult (18,40) (*n* = 257)	TBI‐middle aged (40,64) (*n* = 203)	TBI‐early elderly (64,74) (*n* = 73)	TBI‐late elderly (74,100) (*n* = 42)	Total (*n* = 575)	*p*
Mode of arrival							**0.004**
Ambulance	44 (44.4%)	109 (42.4%)	84 (41.4%)	40 (54.8%)	18 (42.9%)	251 (43.7%)	
Friend/family member	21 (21.2%)	68 (26.5%)	44 (21.7%)	19 (26.0%)	12 (28.6%)	143 (24.9%)	
Self‐transport	24 (24.2%)	71 (27.6%)	66 (32.5%)	8 (11.0%)	6 (14.3%)	151 (26.3%)	
Transfer from other facility	10 (10.1%)	9 (3.5%)	9 (4.4%)	6 (8.2%)	5 (11.9%)	29 (5.0%)	
Missing	0 (0.0%)	0 (0.0%)	0 (0.0%)	0 (0.0%)	1 (2.4%)	1 (0.2%)	
Mechanism of injury							**< 0.001**
Assault or Abuse	2 (2.0%)	31 (12.1%)	12 (5.9%)	0 (0.0%)	0 (0.0%)	43 (7.5%)	
Fall—from standing	53 (53.5%)	56 (21.8%)	91 (44.8%)	56 (76.7%)	32 (76.2%)	235 (40.9%)	
Head struck by/against object	0 (0.0%)	28 (10.9%)	30 (14.8%)	4 (5.5%)	4 (9.5%)	66 (11.5%)	
Motor Vehicle Collision (ejected)	0 (0.0%)	3 (1.2%)	1 (0.5%)	0 (0.0%)	0 (0.0%)	4 (0.7%)	
Motor Vehicle Collision (not ejected)	18 (18.2%)	96 (37.4%)	49 (24.1%)	6 (8.2%)	2 (4.8%)	153 (26.6%)	
Motorized Cycle (with helmet)	0 (0.0%)	5 (1.9%)	3 (1.5%)	0 (0.0%)	0 (0.0%)	8 (1.4%)	
Motorized Cycle (without helmet)	1 (1.0%)	6 (2.3%)	1 (0.5%)	1 (1.4%)	0 (0.0%)	8 (1.4%)	
Other	20 (20.2%)	3 (1.2%)	4 (2.0%)	4 (5.5%)	2 (4.8%)	13 (2.3%)	
Pedal Cycle (non‐motorized with helmet)	2 (2.0%)	0 (0.0%)	3 (1.5%)	1 (1.4%)	0 (0.0%)	4 (0.7%)	
Pedal cycle (non‐motorized without helmet)	0 (0.0%)	1 (0.4%)	0 (0.0%)	0 (0.0%)	0 (0.0%)	1 (0.2%)	
Pedestrian struck by vehicle	1 (1.0%)	2 (0.8%)	5 (2.5%)	1 (1.4%)	1 (2.4%)	9 (1.6%)	
Sports (with helmet)	1 (1.0%)	10 (3.9%)	2 (1.0%)	0 (0.0%)	0 (0.0%)	12 (2.1%)	
Sports (without helmet)	1 (1.0%)	16 (6.2%)	2 (1.0%)	0 (0.0%)	1 (2.4%)	19 (3.3%)	
Presentation from injury (hrs.)	2.8 (1.1–16.7)	1.4 (0.6–12.2)	1.4 (0.7–8.7)	1.6 (0.8–3.6)	2.1 (1.0–8.8)	1.5 (0.7–9.4)	0.18
Was the injury work related?							**< 0.001**
No	88 (88.9%)	220 (85.6%)	166 (81.8%)	72 (98.6%)	42 (100.0%)	500 (87.0%)	
Yes	11 (11.1%)	37 (14.4%)	37 (18.2%)	1 (1.4%)	0 (0.0%)	75 (13.0%)	
First blood draw from injury (hrs.)	13.0 (4.8–26.5)	12.5 (4.2–33.3)	9.2 (4.3–23.0)	8.0 (5.0–23.6)	8.4 (4.5–23.2)	10.0 (4.3–25.8)	0.074
Type of injury							0.28
Blunt Head Trauma		143 (55.6%)	95 (46.8%)	38 (52.1%)	20 (47.6%)	296 (51.5%)	
Blunt Head Trauma with Additional Trauma Injury		114 (44.4%)	108 (53.2%)	35 (47.9%)	22 (52.4%)	279 (48.5%)	
Head neuroimaging results (CT/MRI)							**< 0.001**
Negative		155 (60.3%)	152 (74.9%)	61 (83.6%)	34 (81.0%)	402 (69.9%)	
No CT		88 (34.2%)	34 (16.7%)	2 (2.7%)	0 (0.0%)	124 (21.6%)	
Positive		14 (5.4%)	17 (8.4%)	10 (13.7%)	8 (19.0%)	49 (8.5%)	
GCS							0.41
13		1 (0.4%)	1 (0.5%)	0 (0.0%)	0 (0.0%)	2 (0.3%)	
14		2 (0.8%)	4 (2.0%)	1 (1.4%)	2 (4.8%)	9 (1.6%)	
15		254 (98.8%)	198 (97.5%)	72 (98.6%)	40 (95.2%)	564 (98.1%)	
Headache at intake							0.02
No		38 (14.8%)	41 (20.2%)	24 (32.9%)	11 (26.2%)	114 (19.8%)	
Not Sure		2 (0.8%)	1 (0.5%)	0 (0.0%)	0 (0.0%)	3 (0.5%)	
Yes		217 (84.4%)	161 (79.3%)	49 (67.1%)	31 (73.8%)	458 (79.7%)	
Severe headache							0.69
No		0 (0.0%)	0 (0.0%)	9 (12.3%)	13 (31.0%)	22 (3.8%)	
Yes		0 (0.0%)	0 (0.0%)	5 (6.8%)	4 (9.5%)	9 (1.6%)	
Missing		257 (100.0%)	203 (100.0%)	59 (80.8%)	25 (59.5%)	544 (94.6%)	
Loss of consciousness							0.69
No		139 (54.1%)	109 (53.7%)	37 (50.7%)	28 (66.7%)	313 (54.4%)	
Not Sure		31 (12.1%)	21 (10.3%)	7 (9.6%)	4 (9.5%)	63 (11.0%)	
Yes		87 (33.9%)	73 (36.0%)	29 (39.7%)	10 (23.8%)	199 (34.6%)	
Post traumatic amnesia (PTA)							0.45
No		191 (74.3%)	157 (77.3%)	56 (76.7%)	37 (88.1%)	441 (76.7%)	
Not Sure		10 (3.9%)	4 (2.0%)	1 (1.4%)	1 (2.4%)	16 (2.8%)	
Yes		56 (21.8%)	42 (20.7%)	16 (21.9%)	4 (9.5%)	118 (20.5%)	
PTA (affecting memories before injury)							0.44
No		27 (10.5%)	23 (11.3%)	5 (6.8%)	2 (4.8%)	57 (9.9%)	
Yes		29 (11.3%)	19 (9.4%)	11 (15.1%)	2 (4.8%)	61 (10.6%)	
Missing		201 (78.2%)	161 (79.3%)	57 (78.1%)	38 (90.5%)	457 (79.5%)	
PTA (affecting memories after injury)							1
No		16 (6.2%)	11 (5.4%)	4 (5.5%)	1 (2.4%)	32 (5.6%)	
Yes		40 (15.6%)	30 (14.8%)	12 (16.4%)	3 (7.1%)	85 (14.8%)	
Missing		201 (78.2%)	162 (79.8%)	57 (78.1%)	38 (90.5%)	458 (79.7%)	
Disorientation/confusion							0.03
No		134 (52.1%)	108 (53.2%)	46 (63.0%)	31 (73.8%)	319 (55.5%)	
Not Sure		4 (1.6%)	8 (3.9%)	0 (0.0%)	1 (2.4%)	13 (2.3%)	
Yes		119 (46.3%)	87 (42.9%)	27 (37.0%)	10 (23.8%)	243 (42.3%)	
Focal neurological deficit							0.12
No		159 (61.9%)	140 (69.0%)	55 (75.3%)	29 (69.0%)	383 (66.6%)	
Not Sure		6 (2.3%)	2 (1.0%)	2 (2.7%)	2 (4.8%)	12 (2.1%)	
Yes		92 (35.8%)	61 (30.0%)	16 (21.9%)	11 (26.2%)	180 (31.3%)	
Post traumatic seizure							1
No		251 (97.7%)	199 (98.0%)	72 (98.6%)	42 (100.0%)	564 (98.1%)	
Not Sure		5 (1.9%)	4 (2.0%)	1 (1.4%)	0 (0.0%)	10 (1.7%)	
Yes		1 (0.4%)	0 (0.0%)	0 (0.0%)	0 (0.0%)	1 (0.2%)	

Work‐related injuries occurred in 13.0% of TBI cases overall but were nearly absent in older age groups (*p* < 0.001), reflecting expected age‐related workforce participation patterns.

### 3.6 Hospital Arrival and Imaging Findings

43.7% of TBI patients overall and over half of early elderly participants (54.8%) were transported to the ED by ambulance. Arrival by ambulance increased significantly with age (*p* = 0.004). Younger adults were more likely to self‐transport or arrive with family.

Head imaging findings differed markedly across age groups (*p* < 0.001). Intracranial abnormalities were present in 21.6% of young adults, 17.4% of middle‐aged, 13.7% of early elderly, and 45.8% of late elderly TBI participants, illustrating the increased structural vulnerability of the aging brain at comparable mechanisms of trauma.

### 3.7 Symptoms

Symptom profiles also varied by age. Headache at presentation was reported in 88% of young adults but declined significantly with increasing age (*p* = 0.02). Disorientation or confusion differed by age as well (*p* = 0.03), being more frequent in younger and middle‐aged adults than in older cohorts, which may reflect under‐recognition or baseline cognitive impairment in the elderly.

### 3.8 Biomarkers

**FIGURE 1:**
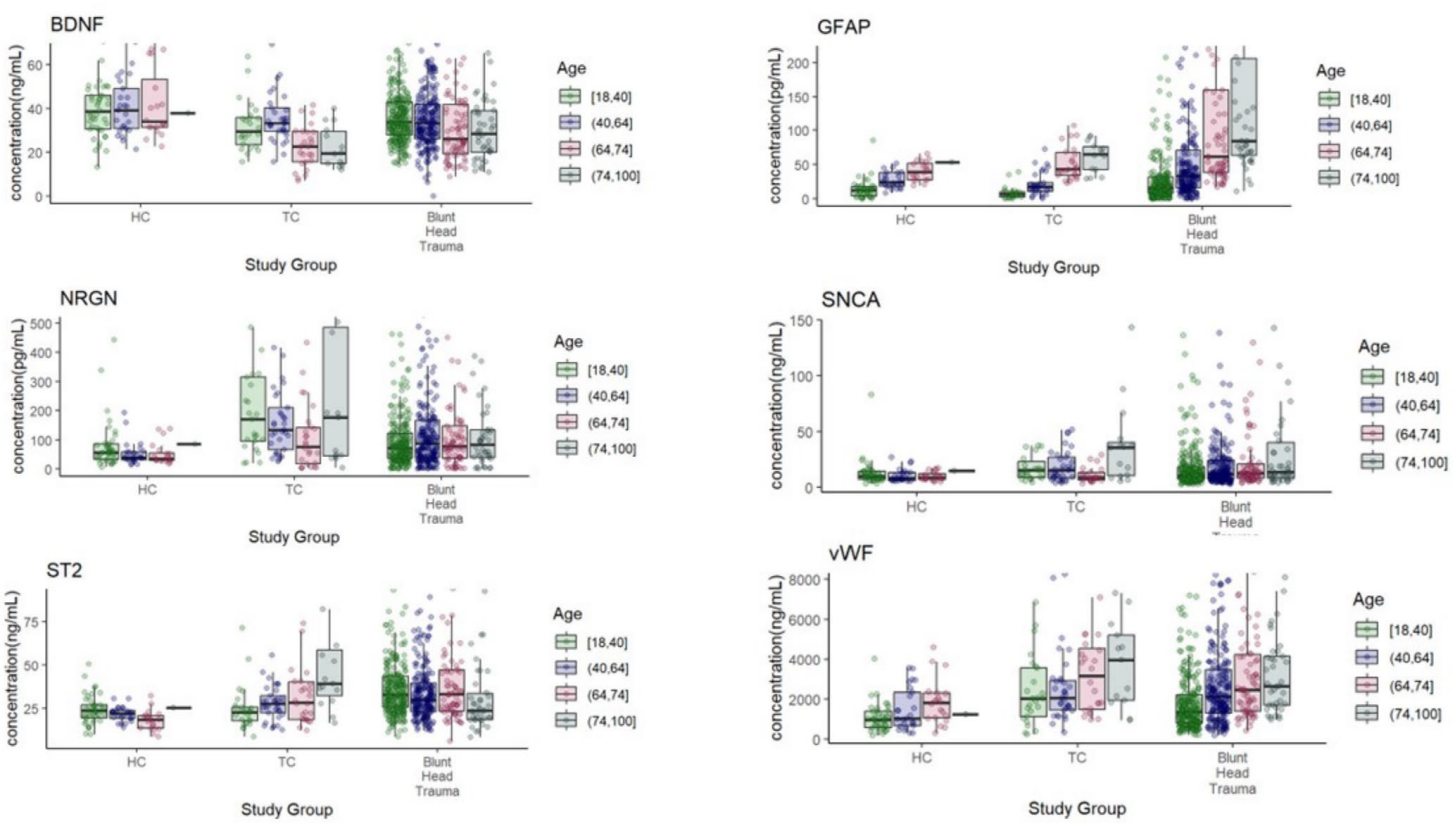
Age‐related differences in glial, neuronal, and vascular biomarkers.

Biomarker distributions across groups are displayed in Figure 1 and summarized in Table 4. Across the combined cohort, distinct quantitative patterns were observed across all markers, with several demonstrating significant associations with age. The full clinical ranges detected for each biomarker protein and group are included in the Table S1.

**TABLE 4:** Effect of age on variance of biomarkers versus injury type.

Biomarker	ANOVA *p*‐value age	ANOVA *p*‐value group	ANOVA *p*‐value age: group	Cohen's *f* age	Cohen's f group	Cohen's *f* age: group
BDNF	< 0.001	< 0.001	0.025	0.22	0.16	0.14
GFAP	< 0.001	< 0.001	0.508	0.49	0.23	0.08
NRGN	0.453	0.001	0.116	0.06	0.14	0.12
SNCA	0.001	0.004	0.157	0.15	0.12	0.11
ST2	0.404	< 0.001	< 0.001	0.06	0.30	0.21
vWF	< 0.001	< 0.001	0.990	0.31	0.23	0.03

Analysis of GFAP levels demonstrated a significant increase with age. Post hoc comparisons revealed that GFAP was higher in participants aged 40–64 compared to 18–40 (*p* < 0.001), in those aged 64–74 versus 18–40 (*p* < 0.001), and in those aged 74–100 versus 18–40 (*p* < 0.001). Comparisons among older age groups showed that 64–74 had higher GFAP than 40–64 (*p* < 0.001) and 74–100 had higher GFAP than 40–64 (*p* = 0.0392), while the difference between 74–100 and 64–74 was not significant (*p* = 0.6704). The interaction between age and group was not significant, indicating that age‐related increases in GFAP were similar across groups. Effect size analysis showed a large effect of age (Cohen's *f* = 0.49), a moderate effect of group (Cohen's *f* = 0.23), and a negligible effect of the interaction (Cohen's *f* = 0.08). These results indicate that GFAP increases robustly with age and is particularly elevated in individuals with TBI, independent of age.

Analysis of NRGN levels indicated that age did not significantly influence protein expression. Age‐related differences were not statistically significant after FDR correction, with only a trend toward higher NRGN in the 64–74 versus 18–40 age group (*p* = 0.058), and all other pairwise comparisons between age groups were non‐significant (*p* ≥ 0.64). The interaction between age and group was also not significant, suggesting that the effect of group on NRGN was consistent across age categories. Effect size estimates indicated a negligible effect of age (Cohen's *f* = 0.06). Overall, these findings suggest that NRGN expression is primarily influenced by group status (Cohen's *f* = 0.14).

Analysis of BDNF levels revealed significant effects of age, group, and their interaction on protein expression. Post hoc comparisons showed that in the 64–74 age group, HC had significantly higher BDNF than TC (*p* < 0.001) and TBI participants (*p* = 0.0036), while TC had lower BDNF than TBI in the same age range (*p* = 0.0123). Age effects were significant overall (*p* < 0.001). Effect size estimates indicated moderate effects of age (Cohen's *f* = 0.22) and group (Cohen's *f* = 0.16), and a small‐to‐moderate effect of the interaction with clinical group (Cohen's *f* = 0.14). These results suggest that BDNF levels are influenced both by age and clinical group, with 40–74 years old participants showing the largest differences between groups, highlighting reduced BDNF after traumatic injury.

Analysis of vWF levels indicated significant effects of age and group, but no significant interaction between these factors. In the 18–40 age group, HC had lower vWF than TC (*p* < 0.001) and TBI (*p* < 0.001), and similar patterns were observed in the 40–64 (HC vs. TC: *p* = 0.0043; HC vs. TBI: *p* = 0.0016) and 64–74 age groups (HC vs. TC, *p* = 0.0168; HC vs. TBI: *p* = 0.0168). Age also significantly influenced vWF overall (*p* < 0.001). Effect size estimates indicated a moderate effect of age (Cohen's *f* = 0.31), a moderate effect of group (Cohen's *f* = 0.23), and a negligible effect of the interaction (Cohen's *f* = 0.03). Overall, these results suggest that vWF increases with age and is higher in TC and TBI compared to healthy controls, particularly in younger to mid‐adult age ranges, with similar age‐related patterns across groups.

Analysis of SNCA levels indicated significant main effects of age (*p* = 0.0014) and group (*p* = 0.0037), while the interaction between age and group was not significant (*p* = 0.1575). Effect size estimates indicated small‐to‐moderate effects of age (Cohen's *f* = 0.15) and group (Cohen's *f* = 0.12), and a small effect of the interaction (Cohen's *f* = 0.11). Overall, these findings suggest that SNCA expression is modestly influenced by both age and clinical group, with the most notable differences observed in the 64–74 age range, where TBI participants exhibited higher SNCA levels relative to both HC and TC.

Analysis of ST2 levels demonstrated a significant effect of group (*p* < 0.001) and a significant age‐by‐group interaction (*p* < 0.001), while age alone did not have a significant main effect (*p* = 0.404). Post hoc comparisons indicated that in the youngest age group (18–40), HC had significantly lower ST2 than TBI participants (*p* < 0.001), and TC also had lower ST2 than TBI (*p* < 0.001), with no difference between HC and TC (*p* = 0.899). In the 40–64 age group, HC again had lower ST2 than TBI (*p* < 0.001). For the 64–74 age group, both HC versus TC (*p* = 0.0002) and HC versus TBI (*p* < 0.001) were significant, whereas TC versus TBI did not reach significance (*p* = 0.0808). In the oldest age group (74–100), TC had higher ST2 than TBI (*p* = 0.0022), but other comparisons were non‐significant. Effect size estimates indicated a negligible effect of age (Cohen's *f* = 0.06), a moderate‐to‐large effect of group (Cohen's *f* = 0.30), and a moderate effect of the interaction (Cohen's *f* = 0.21). Overall, these results suggest that ST2 expression is strongly influenced by group status, with TBI participants generally showing highest ST2 levels. Comprehensive results for ANOVA and effect size analysis are included in Table S2.

The effect of sex on biomarker variance per age group was also addressed in the TBI group (Table S3). For NRGN, the 64–74 age group differed from the 18–40 group, with higher NRGN (Kruskal Wallis, adjusted *p* = 0.008). No other age‐group comparison was significant. For BDNF, 64–74‐year‐old males had a slightly lower BDNF concentration than females (*p* = 0.046). For vWF, 64–74‐year‐old males had a higher serum concentration than females (*p* = 0.0062). Lastly, this analysis showed significantly higher SNCA values in males aged 74–100 years, compared with 18–40‐year‐olds, though this upper age group was small (*n* = 42).

## 4 Discussion

The study findings carry important implications for the clinical translation of biomarkers in mild TBI, as detected clinical ranges differ from younger to older adults. Elevated baseline GFAP, ST2, and vWF could lead to false‐positive interpretations of brain injury unless age‐adjusted [12]. Conversely, age‐related declines in BDNF may obscure pathologic reductions following trauma. Systemic trauma contributes to biomarker variability, as evidenced by elevated NRGN, SNCA, and ST2 in non‐TBI trauma controls. This highlights the need to incorporate both trauma‐specific and age‐specific reference values into diagnostic frameworks.

These results highlight several critical insights. First, the type and mechanism of injury vary substantially across the lifespan, shaping both clinical presentation and biomarker responses. Second, biomarker concentrations in serums differ between trauma‐exposed and head‐injured patients, with distinct profiles observed for glial, neuronal, and vascular markers. Third, age exerts profound effects on biomarker concentrations, often as strong as, or stronger than, injury status. Together, these findings demonstrate that without age‐ and context‐specific reference ranges, biomarker interpretation risks significant misclassification, particularly in older adults.

Demographic patterns reflected well‐known epidemiologic features of trauma and TBI. Socioeconomic and racial differences may influence baseline biomarker levels through mechanisms such as chronic inflammation, cardiovascular risk, and differential comorbidity burden [18, 19]. For example, vWF and ST2 have been associated with cardiometabolic risk and inflammatory states that disproportionately affect historically marginalized populations, while GFAP levels may be influenced by underlying neurovascular and neurodegenerative processes. In contrast, data on racial variation in neuronal biomarkers such as NRGN and SNCA remain limited, though these markers may also be indirectly affected by comorbidity burden and chronic stress exposure. Importantly, these observed differences likely reflect the cumulative impact of structural and environmental factors rather than inherent biological differences, underscoring the need for careful interpretation of biomarker thresholds across diverse populations. Injury features also demonstrated strong age effects: falls predominated among older adults, motor vehicle collisions were most common in middle‐aged groups, and assaults were disproportionately represented among young adults, consistent with prior epidemiologic reports [20]. Importantly, head CT positivity increased sharply in the oldest TBI patients, aligning with evidence of increased structural vulnerability and frailty in the aging brain [3].

Distinct biomarker signatures emerged across study groups. GFAP, ST2, and vWF were consistently elevated in TBI patients compared with both trauma and healthy controls, confirming prior findings [2, 7]. In contrast, NRGN and SNCA were elevated in both trauma and TBI cohorts, suggesting contributions from systemic trauma responses in addition to brain injury [11]. BDNF demonstrated the opposite trend, with the highest concentrations in healthy controls and suppression following both systemic trauma and TBI, consistent with prior work showing BDNF reduction after acute injury and in neurodegenerative disease [21, 22]. These groupwise differences indicate that some markers may be more brain‐specific (e.g., GFAP), while others (e.g., ST2, vWF) capture overlapping systemic and cerebral processes.

Age exerted a substantial and quantifiable influence on multiple biomarkers across all groups. GFAP exhibited the most pronounced association with age, rising steadily from young to late elderly participants. vWF and ST2 followed a similar trajectory, while BDNF declined with increasing age. SNCA showed a smaller but significant upward trend in older adults, and NRGN remained relatively stable. These results align with prior studies demonstrating that advancing age is associated with increased glial reactivity and endothelial dysfunction, accompanied by reduced neurotrophic support [8, 10, 11]. There is still work to be done uncovering the mechanisms behind these age‐related differences, including elucidation of differences in kinetics and clearance for individual markers. Quantitative effect‐size analyses confirmed that the variance explained by age exceeded that of injury status for several analytes, most notably GFAP. Collectively, these findings provide a detailed characterization of biomarker distributions across the adult lifespan and across injury states.

Our findings also support the view that a single biomarker may not be sufficient to characterize a complex injury such as TBI, and it is likely that an algorithmic approach, incorporating multiple biomarkers as well as clinical and demographic variables will be ultimately necessary for biomarkers to be incorporated into management guidelines in brain injury medicine.

Finally, from a research perspective, these results support the development of individualized or age‐adjusted biomarker ranges, particularly for use in acute diagnostic settings [6]. Future studies should validate thresholds in large, demographically diverse cohorts and integrate multimodal data, including imaging, clinical severity, and comorbidity profiles, into predictive models [1]. Longitudinal designs will also be critical to determine whether age‐driven biomarker elevations represent adaptive physiology, subclinical pathology, or heightened vulnerability to poor outcomes.

### 4.1 Limitations

Several limitations should be acknowledged when interpreting these results. Potential confounding factors, e.g., comorbid medical conditions, medication use, and pre‐existing neurodegenerative disease, were not systematically captured. Conditions including chronic kidney or liver disease, diabetes, hypertension, and Alzheimer's pathology can influence biomarker clearance or baseline levels and may have contributed to observed variability. Further unmeasured individual characteristics, including pre‐injury cognitive function, frailty, nutritional status, and substance use (alcohol or drugs), could influence biomarker concentrations and contribute to unexplained variance between groups. Controlling for renal and cardiovascular and renal disease in larger cohorts may also help to better understand the effects of these conditions on biomarkers [23]. These factors warrant systematic evaluation in future studies.

Commonly studied biomarkers such as GFAP have established clinical ranges across multiple immunoassay platforms. However, cross‐platform studies demonstrate variability in signal measurement, highlighting the need for standardized clinical reference materials, which are not yet available for these analytes. While correction factors have been proposed to facilitate comparisons between commonly used instruments, these approaches are limited by differences in assay design, particularly the specific epitopes targeted by binding agents. Despite these challenges, the values observed in the present study are consistent with previously reported clinical ranges, supporting the robustness of our measurements and their relationships to clinical and demographic variables. The observed variability across injury groups and age strata further underscores the importance of multimodal approaches to brain injury characterization, as integrating diverse objective data sources may help account for this heterogeneity and improve diagnostic accuracy.

The cross‐sectional design limits causal inference and precludes assessment of within‐subject biomarker trajectories over time. Measurements were obtained at a single time point within 96 h of presentation; therefore, dynamic post‐injury changes in biomarker levels could not be evaluated. Serial sampling in future work would enable examination of biomarker kinetics, peak timing, and recovery patterns, potentially revealing more nuanced relationships with injury severity and outcome. This study was designed to capture a true ED population, where individuals presented at variable times after a fall. The study was not powered to conduct a sensitivity analysis adjusting for time‐from‐injury, but this is an important future area of research.

Recruitment methods may have introduced selection bias. Healthy controls were enrolled from willing family, siblings, and friends of treated ED patients, which could overrepresent individuals with higher education, socioeconomic stability, and better overall health. In contrast, trauma controls were older and may differ systematically in baseline inflammation, vascular function, or comorbidity burden for comparisons of injury status. These recruitment differences may limit generalizability to the broader population and need further study in large populations. The classification of trauma controls as “non‐TBI” relied on clinical assessment and the absence of reported head impact. Although these criteria are consistent with standard research definitions, occult or subclinical brain injury cannot be entirely excluded, particularly in high‐energy mechanisms such as motor vehicle collisions. Advanced imaging or neurophysiological assessment could help clarify this boundary in future research.

Finally, the study's sample composition and inclusion criteria may not fully capture the diversity of the trauma population. Despite enrollment at Level I trauma centers, the cohorts excluded participants with severe TBI, and few individuals sustained penetrating injuries. Racial and ethnic minority groups remained underrepresented relative to national injury statistics.

### 4.2 Conclusions

In summary, this study demonstrates that both age and systemic trauma strongly influence circulating levels of glial, neuronal, and vascular biomarkers. GFAP, vWF, and ST2 showed marked increases with advancing age, while BDNF declined, and SNCA rose modestly. These age‐associated trends were evident across both trauma and TBI populations and often comparable in magnitude to injury‐related changes. Future longitudinal studies with comprehensive comorbidity profiling, repeated sampling, and multimodal imaging will be essential to clarify the biological mechanisms underlying these relationships and to determine how aging and systemic factors shape biomarker dynamics following injury.

## Author Contributions

R.D.‐A., W.F.P., and D.R.K. contributed to the initial study concept and design. D.S., K.R., R.D.‐A., J.W., W.F.P., D.R.K., and the HeadSMART II investigators contributed to data acquisition. T.E.V.M., N.M., S.B., D.R.K., K.D.M., W.F.P., and R.D.‐A. contributed to data analysis and interpretation and statistical expertise. K.D.M., T.E.V.M., and D.R.K. contributed to drafting the initial manuscript. R.D.‐A., W.F.P., D.S., K.R., S.B., and N.M. contributed to critical revision of the manuscript for important intellectual content.

## Funding

Sample processing and biomarker analyses were provided in kind by BRAINBox Solutions Inc. NINDS grant funding: 1R44NS172072‐A1 was received for HeadSMART Geriatric. No additional external funding was received for this secondary analysis.

## Conflicts of Interest

T.E.V.M., N.M., and S.B. are employed by BRAINBox Solutions Inc., which manufactures a product related to the subject matter. W.F.P. reported receiving grants to Baylor University from BRAINBox Solutions, Beckman, Biocogniv, MeMed, and Werfen. He has received funding personally from Abbott, Biocogniv, Brainbox, Bristol Meyers Squibb, CerebraAI, Oragenics, Osler, Quidel, Radiometer, RCE Technologies, Roche, Siemens, Werfen as a consultant. He owns stock in AseptiScope Inc., Brainbox Inc., Biocogniv Inc., Braincheck Inc., Coagulo Inc., Comprehensive Research Associates LLC, Comprehensive Research Management Inc., Emergencies in Medicine LLC, Prevencio Inc., RCE Technologies, ROMTech, and ScPharma that produces a product relevant to the study material. D.R.K. has received funding personally from and owns stock in BRAINBox Solutions. R.D.‐A. reported receiving grants from BRAINBox Solutions and grants from Abbott to Penn Medicine, nonfinancial support from MesoScale Discoveries (instrument loan and assay support), and personal fees from Danaher outside the submitted work. J.W. has received funding personally from BRAINBox Solutions as a consultant. The other authors declare no conflicts of interest.

## Supporting information


**Figure S1:** Distributions for injury to blood draw duration in blunt head trauma subjects across age groups.

## Data Availability

Research data are not shared.
