# Supplementary material for: Age‐Specific Clinical Biomarker Ranges in Acute Head Injury, Non‐TBI Trauma, and Healthy Control Subjects in the Emergency Department
Source: Acad Emerg Med. 2026 Apr 28;33:e70298. doi: 10.1111/acem.70298 (PMC13123748; doi:10.1111/acem.70298)

Figure S1. Distributions for injury to blood draw duration in blunt head trauma subjects across age groups.

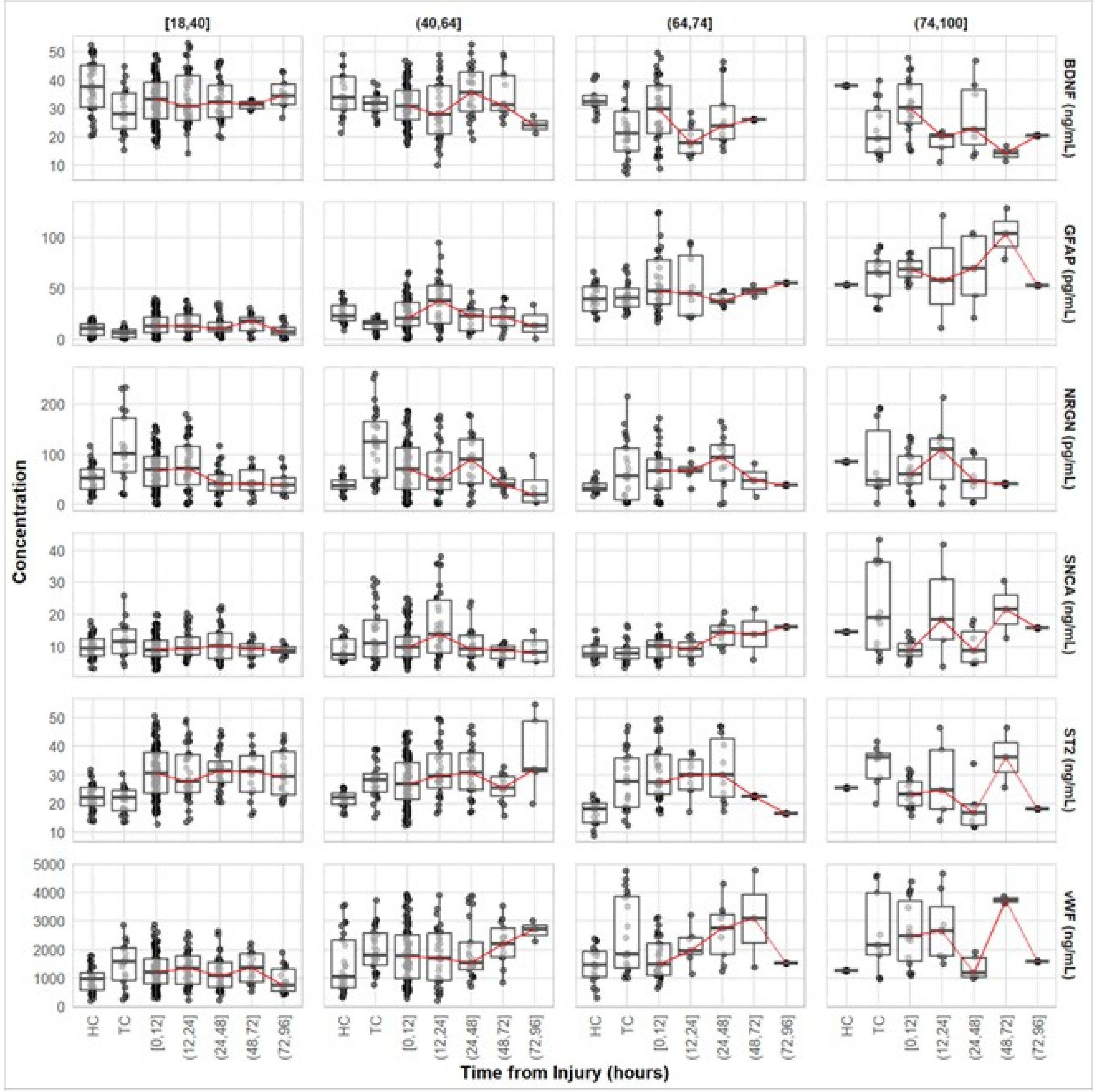

Supplement: Supplementary file 1 — Figure S1: Distributions for injury to blood draw duration in blunt head trauma subjects across age groups. [file ACEM-33-0-s001.pdf]
